# Integrated Approaches to Surveillance of Lymphatic Filariasis and Other Infectious Diseases in the Pacific Islands

**DOI:** 10.3390/tropicalmed11020054

**Published:** 2026-02-14

**Authors:** Adam T. Craig, Harriet L. S. Lawford, Temea Bauro, Clement Couteaux, Litiana Volavala, Myrielle Dupont-Rouzeyrol, Noel Gama Soares, Roger Nehemia, Maria Ome-Kaius, Prudence Rymill, Fasihah Taleo, Patricia Tatui, ‘Ofa Sanft Tukia, Satupaitea Viali, Mary Yohogu, Fiona Angrisano, Leanne J. Robinson, Salanieta Saketa, Andie Tucker, Charles Mackenzie, Susana Vaz Nery, Venkatachalam Udhayakumar, Katherine Gass, Patrick Lammie, Colleen L. Lau

**Affiliations:** 1 Operational Reseach and Decision Support for Infectious Diseases (ODeSI) Program, Centre for Clinical Research, The University of Queensland, Royal Brisbane Hospital, Herston, QLD 4029, Australia; 2 Kiribati Ministry of Health and Medical Services, Tarawa P.O. Box 268, Kiribati; 3 Agence de Santé of Wallis and Futuna, Sia Hospital, Hahake District, Wallis 98600, Wallis and Futuna; 4 Fiji Ministry of Health and Medical Services, Dinem House, 88 Amy Street, Toorak, Suva P.O. Box 2223, Fiji; 5 L’Unité de Recherche et d’Expertise Dengue et Arboviroses, Institut Pasteur de Nouvelle-Caledonia, Nouméa Centre Ville 98800, New Caledonia; 6 Timor-Leste Ministry of Health, Palaco do Governo, Dili TL10001, Timor-Leste; 7 Te Marae Ora Cook Islands Ministry of Health, Tupapa, Rarotonga 0000, Cook Islands; 8 Papua New Guinea Institute of Medical Research, Goroka P.O. Box 60, Papua New Guinea; 9 Vanuatu Ministry of Health, Port Vila P.O. Box 177, Vanuatu; 10 World Health Organization Vanuatu Country Office, Port Vila P.O. Box 177, Vanuatu; 11 Niue Department of Health, Niue Foou Hospital, Alofi P.O. Box 40, Niue; 12 Tonga Ministry of Health, Nuku’alofa P.O. Box 59, Tonga; 13 Oceania University of Medicine, Apia P.O. Box 232, Samoa; 14 Papua New Guinea Ministry of Health, Waigani 121, Port Moresby P.O. Box 807, Papua New Guinea; 15 Vector-Borne Diseases and Tropical Public Health Group, Burnet Institute, Melbourne, VIC 3004, Australia; 16 Public Health Division, Pacific Community, Suva P.O. Box 2223, Fiji; 17 Global Alliance to Eliminate Lymphatic Filariasis, Decatur, GA 30030, USA; 18 Global Health Program, Kirby Institute, UNSW (Sydney), Sydney, NSW 2033, Australia; 19 Task Force for Global Health, Decatur, GA 30030, USA

**Keywords:** infectious diseases, neglected tropical disease, post-validation surveillance, integrated surveillance, serology, serosurveillance, multiplex bead assay, Pacific Islands, Small Island Developing States, health systems

## Abstract

Lymphatic filariasis (LF) is a mosquito-borne neglected tropical disease targeted by the World Health Organization (WHO) for global elimination as a public health problem. Sixteen Pacific Island countries and territories were historically endemic, and eight have now met the WHO criteria for elimination as a public health problem. Elimination as a public health problem does not imply zero transmission. Rather, it denotes that LF prevalence has been reduced below a defined threshold at which community transmission can be sustained. Following validation of elimination, the WHO recommends post-validation surveillance (PVS) to detect potential re-emergence of LF as a public health problem. However, implementing PVS is challenging in Small Island Developing States with dispersed populations, limited workforce capacity, resource constraints, and competing health priorities. The ‘Voices and Visions: Building Partnerships for Integrated Serosurveillance of LF and Other Infectious Diseases in the Pacific Islands’ meeting was held in Brisbane, Australia, from 8–10 July 2025. Fifty-one delegates, including Pacific LF programme managers, WHO representatives, global health partners, and academic researchers, reviewed regional PVS progress, discussed the newly released WHO guidelines for the implementation, monitoring, and evaluation of PVS, planned for PVS implementation, and explored novel multiplex bead assay (MBA) serological analysis methods to strengthen regional coordination for its development as a public health tool. Five broad themes emerged. First, the new WHO *Monitoring and Epidemiological Assessment of Mass Drug Administration in the Global Programme to Eliminate Lymphatic Filariasis: A Manual for National Elimination Programmes, 2nd edn* needs to be operationalised to meet decision-making needs across diverse Pacific settings. Second, integrating LF-PVS with existing surveys and health service activities could improve efficiency and long-term sustainability. Third, regional coordination and alignment of funding cycles will require high-level collaboration. Fourth, community engagement is essential to strengthen demand for PVS. Finally, while at an early stage and with further evidence needed, MBA laboratory methods hold promise for cost-effective, feasible integrated multi-pathogen serosurveillance.

## 1. Introduction

**Figure 1:**
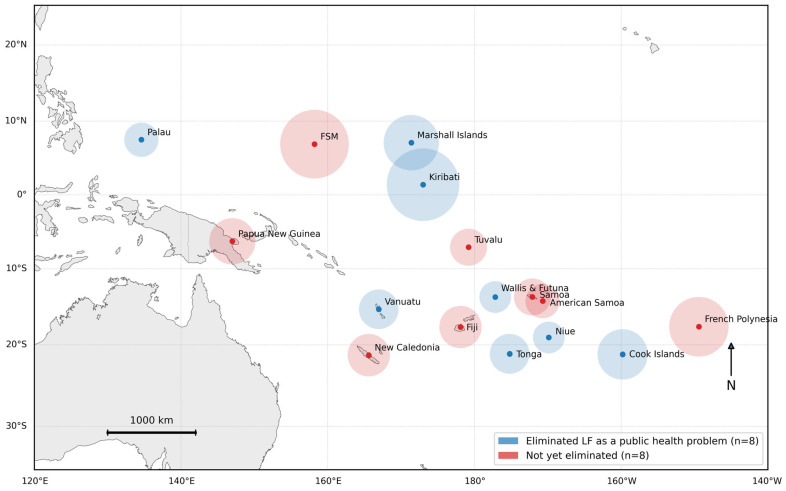
Map of the Pacific region identifying Pacific Island Countries and Territories that have eliminated (blue) and not yet eliminated (red) lymphatic filariasis as a public health problem, 2026. Highlighted areas are illustrative ocean footprints to improve visibility of small islands.

Lymphatic filariasis (LF) is a mosquito-borne parasitic neglected tropical disease (NTD) that damages the lymphatic system and can lead to lymphoedema and hydrocele, conditions associated with lifelong disability and social stigma [1]. Through the Pacific Programme to Eliminate Lymphatic Filariasis (PacELF) [2], eight of the sixteen Pacific Island countries and territories (PICTs) that were endemic for LF (Cook Islands, Kiribati, Niue, the Republic of the Marshall Islands, Palau, Tonga, Vanuatu, and Wallis and Futuna) have reached World Health Organization (WHO) targets and have been validated as having eliminated the disease as a public health problem (Figure 1; Table 1). WHO describes the elimination of LF as a public health problem as when the prevalence target threshold of <1% antigen (*Ag*) (*W. bancrofti*) and antibodies (*Ab*) (*Brugia* spp.) for all vector and parasite species is reached [3,4]. This does not equate to zero transmission of LF, as the presence and transmission of the parasite may persist at low levels. WHO therefore recommends post-validation surveillance (PVS) for LF to detect and respond to re-emergence early and safeguard progress [3].

PVS involves the systematic collection and interpretation of epidemiological, entomological and/or environmental data after the WHO validates LF elimination as a public health problem [3]. Its purpose is to identify residual or new infections before widespread community transmission re-emerges. In PICTs and other small island States and Territories, PVS is complicated by highly dispersed populations, logistical constraints, workforce shortages, limited infrastructure, and competing public health demands [5,6,7].

The ‘Voices and Visions: Building Partnerships for Integrated Serosurveillance of LF and Other Infectious Diseases in the Pacific Islands’ (hereafter referred to as the “Voices and Visions meeting”) was held from 8 to 10 July 2025 at The University of Queensland, Brisbane, Australia. Fifty-one participants attended, including national LF programme managers from 11 PICTs (Table 1), representatives from WHO, global LF advisors and research partners. The meeting aimed to: (1) support peer learning by sharing experiences of LF PVS implementation; (2) interpret and contextualise the newly released WHO *Monitoring and epidemiological assessment of mass drug administration in the global programme to eliminate lymphatic filariasis: a manual for national elimination programmes, 2nd edition* [3] (hereafter referred to as the “PVS M&E Guidelines”); (3) identify opportunities to integrate LF surveillance with other health programmes; (4) strengthen understanding of *Ab*-based tools as potentially more sensitive alternatives to *Ag* detection for LF surveillance; and (5) explore cost-effective approaches to integrating multi-pathogen serological surveillance with existing programmes.

**Table 1:** Overview of lymphatic filariasis elimination status and most recent epidemiological surveillance findings across Pacific Island Countries and Territories.

Pacific Island Country or Territory	Population Est in 2026	Year Eliminated LF as a Public Health Problem	Year of the Most Recent Published LF Study	Summary of the Most Recent Published Study Findings *	Representative at the Voices and Visions Meeting
*Countries that have eliminated LF as a public health problem*					
Cook Islands ^%^	15,406	2016	2013–2014 [8]	A total of 2903 participants from ten islands were tested. Only one individual was *Ag*-positive for LF, and no additional *Ag*-positive people were identified across the remaining 11 islands. The national *Ag* prevalence was estimated as being 0.23%.	Yes
Niue ^^^	1543	2016	2009 [9]	A whole-population survey (n = 1378) reported an overall LF *Ag* prevalence of 0.5%, with no positive cases detected among six- to seven-year-old children.	Yes
Vanuatu ^^^	307,941	2016	2010–2012 [10]	A total of 4480 school-aged children were screened across three sampling units, with no *Ag*-positive cases detected. A subsequent transmission assessment survey in one unit identified two *Ag*-positive children among 933 tested. The resulting national *Ag* prevalence was estimated at 0.2%.	Yes
Wallis and Futuna ^%^	11,151	2018	2025 [11]	A cross-sectional study of 353 schoolchildren aged 18 years or younger identified five *Ag*-positive cases, corresponding to an estimated antigenemia prevalence of nearly 2% in Futuna. The investigation also confirmed a spatial cluster where antigenemia prevalence reached 7.5% (95% CI: 2.1–18.2%). Follow-up assessments detected microfilariae (Mf) in several contact cases.	Yes
Palau	17,976	2017	2001 [12]	A baseline assessment of 2031 people conducted in 2001 identified nine *Ag*-positive cases from the same village, giving an *Ag* prevalence of 0.4%.	No
Tonga	99,283	2017	2024 [13]	A survey of 1787 participants recruited from 12 communities, 11 primary schools, five high schools, and one outpatient clinic identified 39 *Ag*-positive cases (2.2%) and five Mf-positive cases (0.3%). The community with the highest burden recorded an *Ag* prevalence of 4.0% (95% CI: 2.9–5.6%).	Yes
Republic of the Marshall Islands	54,446	2017	2003 [12]	A 2001 baseline survey of 2003 people across two islands identified 2 *Ag*-positive cases, an *Ag* prevalence of 0.1%. In 2002, follow-up blood *Ag* surveys reported 130 *Ag*-positive individuals among 294 people on Mejit (44.2%) and 71 positive people among 244 people on Ailuk (29%). By contrast, similar surveys conducted in 2003 found no *Ag*-positive individuals among the 217 people examined on Wotje Atoll or 318 examined on Ebon Atoll.	No
Kiribati ^^^	122,735	2019	1999–2000 [12]	A baseline assessment A survey found an *Ag* prevalence of 1.7%.	Yes
*PICTs that have not yet eliminated lymphatic filariasis as a public health problem*					
American Samoa	57,085		2016 [14,15]	Among 2671 survey participants, 135 tested *Ag*-positive, giving an overall *Ag* prevalence of 5.1%. These findings confirmed ongoing LF transmission in previously recognised clusters and hotspots and highlighted new areas that merit further investigation.	No
Fiji	901,603		2007 [16]	A nationwide stratified cluster survey reported an *Ag* prevalence of 9.5%, ranging from 0.9% in the Western Division to 15.4% in the Eastern Division. Mf prevalence was 1.4%.	Yes
French Polynesia	280,855		2008 [17], 2021 [18]	LF transmission persists in several island groups, including the Society Islands, the Southern Marquesas, and the Gambier Archipelago. A 2008 cross-sectional, stratified three-cluster survey of 1178 people aged ≥2 years reported an ICT-positive prevalence of 11.3% and a Mf prevalence of 10%. Complementing these findings, a molecular xenomonitoring study of 5508 female mosquitoes collected from 420 sampling points on Huahine Island (within the Society Islands) detected LF-positive vectors in 13 of 28 primary sampling units.	Yes
Federated States of Micronesia	105,987		2003 [19]	A survey of 233 participants on Satawal Island found 96 *Ag*-positive cases (38%), and 55 individuals (22%) with circulating Mf.	No
New Caledonia	274,330		2013 [20]	A survey of 1035 participants identified seven *Ag*-positive cases. All participants were negative on Mf blood smears and for filarial deoxyribonucleic acid (DNA). The overall *Ag* prevalence was 0.62% (95% CI: 0.60–0.63). Although this is below the WHO threshold for elimination of LF as a public health problem, the absence of clear epidemiological evidence excluding domestic transmission has led health authorities to consider the possibility of ongoing circulation.	Yes
Papua New Guinea	9,311,874		Multiple years [21,22,23,24]	Surveys conducted in East Sepik and Sandaun Provinces between 2013 and 2018 (n = 2854) report Mf prevalence of 24.0% (95% CI: 22.9–26.1) and *Ag* prevalence of 46.4% (95% CI: 43.5–49.3) at baseline declining to 0% Mf and 29.5% Ag prevalence (CI, 26.9–32.3%) after yearly treatment for 3 years. Surveys conducted in Bogia, Madang Province in 2016–2017 (n = 2382), reported Mf prevalence of 4 × 4% (95% CI: 3.6–5.3) and *Ag* prevalence of 22.0% (95% CI: 20.2–23.6) at baseline declining to 0 × 4% Mf (CI: 0 × 1–0 × 7) and 16.3% (95% CI: 14.9–17.9) *Ag* prevalence 12 months after one round of IDA, and further declining to 0 × 2% Mf (95% CI: 0.1–0.5) and 7.5% (95% CI: 6.4–8.7) *Ag* prevalence at 24 months (after a second round of IDA). A summary of older surveys by Graves et al. [21] reported LF prevalence ranges of 30.4–64.7% between 1983–1992, 30.1–56.9% between 1993–2000, and 7.8–12.8% between 2003–2011.	Yes
Samoa ^^^	200,999		2023 [25]	A survey of 623 participants aged five or older from 125 randomly selected households across eight sampling units identified *Ag*-positive cases in six of the units. The adjusted *Ag* prevalence was 9.9% (95% CI: 3.5–21.0).	Yes
Tuvalu	10,778		2007 [16]	A whole-population serosurvey identified 973 *Ag*-positive individuals among 8173 people tested, giving an *Ag* prevalence of 11.9%.	No

* The information presented does not provide a comprehensive history of lymphatic filariasis in Pacific Island Countries and Territories. It does not document mass drug administration activities, C-survey or transmission assessment survey findings, nor does it include results from programme monitoring and evaluation activities that may have been undertaken but not yet published. ^^^ Countries where surveys have recently been conducted, but results are not yet published. ^%^ Countries and territories that plan to conduct lymphatic filariasis monitoring and evaluation surveys in 2026.

## 2. Results and Discussion

Data informing this report were drawn from a thematic review led by ATC, with contributions from all co-authors. Sources included artificial intelligence–generated transcripts of meeting discussions, content from presenters’ slides, outputs from interactive workshops, and facilitators’ and presenters’ notes. Consensus on the themes and reported content was achieved through iterative review by the co-authors, all of whom were meeting participants. The same iterative process was used to validate the final content.

**Figure 2:**
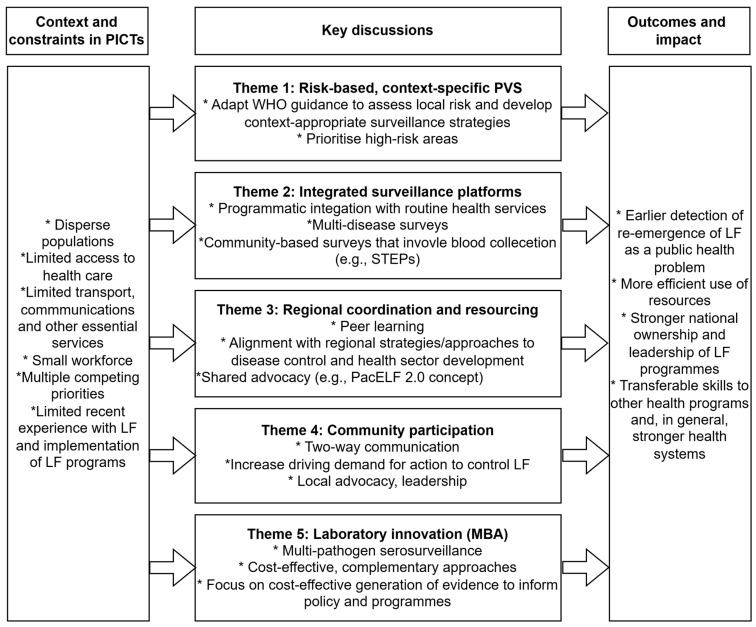
Overview of the key themes and discussions at the ‘Voices and Visions: Building Partnerships for Integrated Serosurveillance of Lymphatic Filariasis and Other Infectious Diseases in the Pacific Islands’ meeting, Brisbane, Australia, 8 to 10 July 2025.

This report does not present the proceedings in chronological order; instead, it synthesises and reports key discussions over the three days of the meeting into a set of overarching themes. Figure 2 presents these themes graphically, and the subsequent sections provide detailed results and discussion.

### 2.1. Theme 1: Context-Specific Approaches to Post-Validation Surveillance

The PVS M&E Guidelines [3] are intentionally flexible, acknowledging the wide variation in epidemiological risk and health system capacity across the globe. Participants reached an early consensus that, while the PVS M&E Guidelines provide a valuable foundation, they must be interpreted and adapted to meet the diverse, unique, and complex needs of PICTs. Several delegates initially expressed a desire for greater clarity and a more prescriptive, checklist-style approach. However, discussions throughout the meeting reinforced that PVS should be adaptive, risk-based, and firmly grounded in local realities.

Examples shared during the meeting highlighted how rigid surveillance models can prove to be impractical. Tonga described the challenges encountered in conducting the LF Transmission Assessment Surveys, which required complex, large-scale school-based sampling but yielded limited actionable insights. The delegate from Samoa added that applying uniform sampling approaches can overburden health authorities while under-representing areas of higher transmission risk. Delegates concluded that adopting a risk-based approach to PVS offers a more efficient, context-appropriate strategy. However, participants acknowledged the challenges of defining risk strata where data on past LF prevalence are incomplete or outdated. In such situations, delegates agreed that conducting a risk assessment that integrates historical evidence of transmission with current surveillance data and local knowledge offers the most practical and context-appropriate foundation for PVS design [13].

Community engagement was repeatedly emphasised as central to effective PVS implementation. Delegates from the ministries of health in Tonga and Niue observed that when communities understand the purpose and value of PVS, participation and data quality improve. Conversely, poorly explained or overly complex and burdensome surveillance activities can exacerbate survey fatigue and, as described by a participant from Samoa, erode trust, an issue that has become more widespread since the COVID-19 pandemic [26,27]. As one Pacific health manager aptly put it, “Surveillance should be about listening to our communities and learning from them, not just ticking boxes.”

The absence of highly prescriptive guidance raised questions about accountability and comparability across countries. To balance flexibility with consistency, participants proposed establishing a core data package for PVS reporting in the Pacific. Suggested indicators included the frequency and scope of surveys, the number of localities sampled, and the proportion of circulating filarial *Ag*-positive results. Collecting and reporting these indicators through existing national and regional systems was viewed as a pragmatic step to strengthen accountability, sustain momentum, and enable regional benchmarking of PVS progress.

In practice, a risk-based approach to PVS could be operationalised by prioritising geographic areas based on a combination of historical LF prevalence, time since the last MDA, recent surveillance findings, and local programmatic knowledge. For example, programme managers may prioritise intensive or frequent surveillance in communities with previously documented hotspots or recent antigen-positive cases, while applying a more conservative “light touch” approach in areas considered low risk. This approach allows limited resources to be targeted where the likelihood and consequences of resurgence are greatest, while remaining aligned with WHO guidance.

### 2.2. Theme 2: Integrating LF-PVS with Established Surveys and Routine Health Service Programs

Participants felt that programmatic integration has the potential to offer a viable, sustainable approach to PVS and to strengthen broader surveillance capacity and health systems. Participants emphasised that given the financial, human, and time resources required, the independent implementation of PVS is, at this point, unrealistic for most PICTs. They also noted that tasks involved in LF surveys (i.e., blood collection and questionnaires) are duplicated by other activities and suggest that integrating LF surveillance into existing data collection platforms, such as non-communicable disease (NCD) surveys, surveys of other NTDs, demographic and health surveys, vaccine-preventable disease surveys, and public health services such as diabetes clinics or peri-natal clinics, where blood is routinely collected offers opportunities for cost and labour saving, making PVS more feasible.

Several countries shared successful experiences of integration. Niue, for example, reported leveraging a planned and funded WHO STEPwise approach to NCD Risk Factor Surveillance survey to collect blood samples and demographic information for LF-PVS [28], Tonga showcased integration with routine health facility-based delivery of care [13] and delegates from Papua New Guinea, where malaria and LF share vectors and areas of endemicity, advocate for the unique opportunity to coordinate surveillance and vector control activities to address both diseases synergistically [29]. These examples illustrate the feasibility of integration and provide several practical models that programme managers may consider adopting.

Integration was also discussed beyond field surveys. Participants noted that facility-based surveillance systems, such as testing patients at a diabetes outpatient clinic in Tonga [13], can be leveraged to implement PVS. Furthermore, digital surveillance platforms that integrate data across diseases, such as ‘District Health Information Systems, version 2’ (DHIS2), can serve as a common repository for both LF and other infectious diseases.

Integration is not without challenges. Delegates note that different programmes typically operate on separate funding cycles, have distinct reporting schedules, and pursue different goals and operational requirements, including sampling design and collection approaches. Harmonising these elements across programmes requires high-level coordination among multiple partners, which takes time and often lacks the trust needed. Delegates therefore recommended advocating for integration through the lens of operational and cost-efficiency within national health planning, and, using LF-PVS as an example, identifying integration opportunities and aligning schedules.

### 2.3. Theme 3: Regional Coordination and Resourcing to Support PVS

During the meeting, participants reflected on the early success of PacELF, which provided a coordinated, structured, and regional approach to LF elimination as a public health problem. This initiative fostered strong political commitment, regional solidarity, and substantial public health gains [2]. With the conclusion of PacELF, delegates noted that structured programmatic support has diminished, leaving PICTs with limited access to funding, technical expertise, and advocacy mechanisms to sustain momentum for pre-validation and PVS activities. The decline in regional coordination was viewed as contributing to reduced political attention and prioritisation of LF elimination as a public health problem.

To address this gap, participants proposed establishing a revitalised regional mechanism, informally referred to as ‘PacELF 2.0,’ to reinvigorate a coordinated regional approach to eliminating LF as a public health problem. The proposed mechanism would (i) advocate for continued political commitment and financial investment to achieve LF elimination as a public health problem across the Pacific; (ii) facilitate regional peer-to-peer learning and development of good practice guidance; (iii) prioritise and commission operational research, and support quality improvement initiatives; and (iv) coordinate regional LF surveillance and response activities.

Delegates from countries where LF has been eliminated as a public health problem highlighted the importance of (and challenge in sustaining) financial investment to operationalise PVS, noting that funding for LF elimination as a public health problem has declined. In practice, participants emphasised the need for predictable financing (whether from international or domestic sources) to plan PVS activities. Partnerships with regional and global organisations were seen as key to both mobilising resources and ensuring LF, as a neglected disease, remains on the development agenda.

### 2.4. Theme 4: Promoting Community Participation in PVS

Participants consistently emphasised that technical excellence alone is insufficient to sustain LF elimination as a public health problem; the long-term success of PVS depends on communities’ understanding, trust, and demand for surveys.

Delegates noted that in many PICTs, surveillance is perceived as an externally driven activity, disconnected from community priorities and providing little visible benefit [30]. Several participants reflected that after years of MDA and external surveys, communities in pre-LF elimination as a public health problem countries are now experiencing “survey fatigue” and may question the value of repeated testing. Meeting participants agreed that for PVS to be accepted, it must move beyond data extraction toward the co-creation of knowledge valued by both health authorities and communities alike.

Delegates stressed that communication must be two-way and culturally grounded. Community members should not only be informed about surveillance activities but also invited to help design them. Participants noted that providing feedback on results is equally important, as communities are more likely to remain engaged when they see how their contributions lead to action. “People give blood because they care about their village,” one delegate noted, “but they deserve to know what their blood is telling us.”.

The role of trusted intermediaries, such as village health committees, church leaders, and women’s groups, was identified as essential to bridging technical and community perspectives. Delegates agreed that these actors can translate scientific information into locally meaningful messages and foster dialogue about disease risk and prevention. Discussions further underscored that meaningful participation requires capacity building at the community level. Training local health workers and community advocates to collect samples, interpret basic data, and communicate findings can transform LF surveillance from a perceived top-down, externally driven activity into a shared local responsibility.

From an operational perspective, community engagement may be embedded into PVS by involving local leaders and community health workers in survey planning, sample collection, and the communication of results. Such approaches will likely help shift any perception that PVS is an externally driven exercise toward a locally valued public health activity.

### 2.5. Theme 5: Leveraging Multiplex Serological Tools for Integrated Surveillance

The meeting also discussed the use of the new MBA analysis using Luminex^®^ technology to detect *Abs* (immune markers usually indicative of past exposure or immunity) for a panel of infectious diseases, including two *Abs* for LF (anti-*Wb123* and anti-*Bm14*) and its potential for LF surveillance [31].

Discussions explored both the benefits and challenges of using the MBA in the Pacific context. Participants highlighted opportunities for programmatic integration and cross-programme cost-sharing, but also noted several constraints, including limited access to testing facilities, the high costs that currently limit its feasibility as a public health tool, the reliability of the supply of laboratory consumables needed for analysis (particularly *Ag*-coupled beads), and the need for validation of results across time and between laboratories.

It was stressed that *Ab* testing for LF is still a developing tool, and results should be interpreted with a high degree of caution. WHO’s PVS M&E Guidelines state, “*…exposure to filarial parasites may induce antibodies in people, even if a true infection does not occur. Infected people, both microfilaraemic and amicrofilaraemic, have elevated levels of antibodies, but the results of antibody testing do not distinguish between current and past infection. Nevertheless, detection of antibodies in children demonstrates recent exposure to filarial parasites* [3].” Delegates concluded that while *Ab* data offer opportunities, at this stage, they should be interpreted alongside other established indicators, such as *Ag* and Mf, to avoid false signals of LF re-emergence or missed transmission events. Transparent communication about uncertainty, particularly in the early stages of technology implementation, was deemed essential to build understanding of MBA and its limitations and to maintain stakeholders’ engagement and trust in the development process.

Discussions then shifted to how MBA development could be grounded in the realities experienced by PICT public health decision-makers. Delegates emphasised the importance of health consumers (i.e., the public) and knowledge end-users (i.e., PICT ministries of health) in establishing MBA as a public health tool. To foster coordinated regional engagement, participants discussed establishing a network to advance thinking about how integrated multi-pathogen serosurveillance may be used in the Pacific. Representatives from Ministries of Health, academic institutions, laboratories, and partner organisations expressed support for this initiative, which has been named the Serosurveillance Partnership for the Pacific Region (SERO-PAC). Positioned as “a platform through which integrated surveillance can become a sustainable long-term approach to disease monitoring throughout the [Pacific] region,” SERO-PAC aims to build a collaborative, locally led, and regionally coordinated mechanism capable of generating reliable reagents and resources, strengthening analytical capacity, and supporting the integration and use of multi-pathogen serosurveillance data [32].

Participants also highlighted several cross-cutting challenges that may constrain the implementation of LF surveillance. In many PICTs, historical LF data are incomplete, outdated, or not available across all geographic areas, complicating risk stratification and the targeting of surveillance activities. Financial constraints remain a major barrier, with limited domestic resources and declining external funding, reducing the feasibility of stand-alone surveys or repeated testing. While MBA–based serology offers promise for integrated and cost-efficient surveillance in the longer term, participants noted that its current feasibility is limited by high per-sample costs, restricted access to specialised laboratories, supply chain vulnerabilities, and the need for further refinement of analytical methods to ensure results provide clear insights relevant to policymakers’ information needs. Delegates emphasised that new approaches to LF surveillance should be introduced cautiously, align with programmatic needs and health system capacity.

## 3. Conclusions

The ‘Voices and Visions’ meeting reaffirmed the Pacific region’s ongoing commitment and leadership in sustaining LF’s elimination as a public health problem. It also demonstrated collective determination to harness innovation, such as integrated surveys and multiplex serological tools, to address broader health system challenges that impede infectious disease surveillance and control. Discussions catalysed new ideas and collaborations that will shape the next phase of LF-PVS across the Pacific. Sustained regional coordination, policy support and investment from partners are crucial to translating these ideas into practice. To maintain the momentum of the meeting, delegates emphasised the value of periodic follow-up activities, with plans for both online and face-to-face engagements underway. Delegates further committed to lobbying national health leaders to support and implement LF-related actions.

## Data Availability

Data are available from the corresponding author upon reasonable request.

## Abbreviations

The following abbreviations are used in this manuscript:

*Ab*	Antibodies
*Ag*	Antigen
CI	Confidence interval
COR-NTD	Coalition for Operational Reseach on Neglected Tropical Diseases
COVID-19	Coronavirus-19
ENGAGE	Ending the Neglect through Greater Access and Greater Effectiveness
GAELF	Global Alliance to Eliminate Lymphatic Filariasis
HERA	Health Research Accelerator (program of The University of Queensland)
NHMRC	National Health and Medical Research Council
NTD	Neglected-tropical disease
LF	Lymphatic filariasis
M&E	Monitoring and evaluation
MBA	Mulitplex bead assay
Mf	Microfilaria
NCD	Non-communicable disease
PacELF	Pacific Programme to Eliminate Lymphatic Filariasis
PICTs	Pacific Island Countries and Territories
PVS	Post-validation surveillance
SERO-PAC	Serosurveillance Partnership for the Pacific Region
STEPs and STEPwise	“STEPs” and “STEPwise” refer to the step-by-step structure of the WHO NCD risk factor survey methodology rather than being an acronym
UQ	The University of Queensland
WHO	World Health Organization
